# HR practices and turnover intention; the mediating role of organizational commitment in Tehran: a cross-sectional study

**DOI:** 10.12688/f1000research.73351.2

**Published:** 2022-09-22

**Authors:** Mohammaad Kashinejad Hassanpour, Chin Wei Chong, Siong Choy Chong, Mohammad Khaleel Ibrahim Okour, Samadi Behrang, Xin Yee Tan

**Affiliations:** 1 Faculty of Management, Multimedia University, Cyberjaya, Selangor, 63100, Malaysia; 2 Senior Office, Finance Accreditation Agency, Kuala Lumpur, Kuala Lumpur, 50480, Malaysia; 3 Business Administration, Luminus Technical University College, Amman, 11118, Jordan; 4 Assistant Professor of Management, Business Administration Department,, Amman, Jordan; 5 School of Marketing and Management, Asia Pacific University, Kuala Lumpur, Kuala Lumpur, 57000, Malaysia

**Keywords:** HR practices, turnover intention, organisational commitment, learning organisations, renewable energy industry

## Abstract

**Background:** Employees are increasingly being recognised as a valuable source of information, especially in knowledge-based businesses. Businesses, however, suffer financial and organisational memory losses related to re-hiring and training new staff, and lost productivity and intellectual property because of employee turnover. Hence, employee turnover should be considered an essential part of human resource management. Furthermore, employees’ trust in management and human resource (HR) practices substantially impact organisational commitment (OC). Thus, anticipating employee commitment and turnover intentions is crucial, as people are the sole source for knowledge-based firms to maintain their competitive advantage. In the context of selected Tehran Renewable Energy (RE) firms, this study investigated the mediating impact of OC on the relationship between HR practices (recruitment and selection; training and development opportunities; performance appraisal and evaluation; teamwork; compensation and pay; and job security) and employee turnover intention.

**Methods:** This is a cross-sectional study in Tehran that involved 90 experts and knowledgeable employees from four of Tehran's top RE businesses. A questionnaire was distributed to collect data which was later analysed with correlation, regression and bootstrapping analyses.

**Results:** All six dimensions of HR practices were discovered to have an indirect impact on turnover intention and a direct impact on OC. OC among employees has an indirect effect on turnover intention. It was also revealed that the training and development opportunity has the most considerable effect on OC and turnover intention. OC was not found as a mediator between HR practices and turnover intention.

**Conclusions:** The outcomes of this study showed that both training and development opportunities; and pay and compensation structure were found to be two significant components of HR practices in the relationship with OC. RE managers should employ appropriate HR strategies, particularly in these two dimensions, to improve an individual's degree of OC and reduce turnover intention.

## Introduction

Talent management has become more critical as part of companies' strategies to remain competitive by attaining organisational effectiveness. Employers, however, risk losing employees who seek better opportunities elsewhere. Therefore, employers must establish talent management systems that help develop talents and, most importantly, retain personnel to counter the loss of well-trained and experienced personnel. Massive investments in shaping and developing human capital will be nought if staff quit and take their talents and experience to other companies.

The renewable energy (RE) field is recognized both as a technological- and knowledge-based industry by which its employees play a vital role. As elucidated by Salehi-Isfahani (2005), ^28^ human capital in this industry can be used as a source of competitive advantage because they are scarce and inimitable. The experts in this industry are thus an important competitive component and that the RE industry must concentrate on retaining its employees. However, the RE companies in Tehran have been experiencing high employee turnover. Three probable reasons have been identified. Most of them desire for a change in workplace. Some of them migrate to other countries to work in a more established RE sector. There is also a fraction of those who leave to set up their own companies. There are relatively fewer experts in this field in Tehran and therefore, the RE organizations are finding it challenging to find competent replacements when employees leave.

Losing good employees can impair an organisation's competitive advantage by diminishing production and quality. Demographics, satisfaction, organisational commitment (OC), and human resource management (HRM) practices having been identified as significant factors influencing turnover intention. OC has been found to impact turnover intention considerably. Therefore, it appears that OC must be included to forecast turnover. Human resource (HR) practices are also reported to affect turnover intention, however, with limited empirical research. Therefore, the need to study factors influencing turnover intention are pertinent to provide more precise insights on how companies can retain their employees. The goals of this study were to look at the impacts of HR practices on OC and turnover intention and investigate if OC may mediate the relationship between HR practices and turnover intention.

### The effects of HR practices on organisational commitment

HR practices are how companies shape employee behaviour, perception, and attitude. ^33^^,^^36^ HR practices like development opportunities, performance evaluation, and compensation have been investigated extensively, and significant impacts on OC were reported. ^2^ Organisations should achieve their goals primarily dependent on human capital if HR practices are appropriately designed and implemented. As human capital nowadays becomes the primary asset in organisations, managing them to make them perform and think in the ways employers desire is pertinent. The level to which employees are willing to exert extra effort for the company’s success and the degree of alignment between employee and company values is measured by OC. ^19^ HR practices that directly influence employees' OC significantly influence company success, and OC is an important criterion to measure HRM effectiveness. ^2^^,^^34^ Employees interpret good HR practices positively and reciprocate with high OC. ^3^

Examining the impact of HR practices on OC requires a closer look at HRM elements. Because of their unique characters, each factor should be investigated separately because it may have a different impact on OC. Ideally, HR practices should be integrated and intertwined. They should be regarded as a single entity, but some organisations have yet to develop systematic HR practices and implement them, so examining individual HR practices is still necessary. ^13^

Following a review of the research and evaluation of the environmental and cultural circumstances within the RE business in Tehran, six HR practices were identified by the authors as critical antecedents to employee OC:

*Recruitment and selection.* The recruitment operations and selection process aim to recruit and appoint the best candidate for the vacancy. It refers to the employment of potential human capital which includes identifying, assessing, attracting capable applicants, and allocating most suitable individuals to jobs. It usually begins with the job description anends with the applicant’s appointment. Hiring the right individual increases organisational productivity, with lesser performance issues and greater job satisfaction, translating into long-term OC and, more importantly, a lower tendency to quit. ^8^^,^^22^

*Training and development opportunities.* The influence of a company's focus on training opportunities on turnover intention was found contradictory. It was asserted that training is an essential aspect that positively and significantly influences an employee's attitude and the efficacy and productivity of an organisation, reducing turnover. ^22^^,^^26^^,^^29^^,^^31^ Organizations with advanced development programs are expected to face fewer employees leaving compared to organizations which do not consider employee-related programs. However, several studies also suggested that training may not significantly impact OC; hence it’s worth investigating. ^3^^,^^15^^,^^32^

*Performance appraisal and evaluation.* It is a designed system that evaluates employee’s behavior related to their assigned jobs and is used to facilitate decision making by employers such as compensations, lay-offs, and promotions which may increase effectiveness. Employee performance is assessed based on several factors, and if performance evaluations are poorly designed and executed, employees will fail to recognise the value of such exercises. ^22^ This situation could arise because of a faulty link between performance appraisal and compensation or because employees have lost faith in performance appraisal, ^32^ resulting in lower OC and higher turnover. ^2^^,^^4^

*Teamwork.* The term “teamwork” refers to a group's coordinated actions, and it directly impacts employee OC. Employees who work in teams feel more fulfilled and accomplished at work, which leads to increased employee retention. ^36^

*Compensation and pay.* Fair compensation methods have been demonstrated to have several advantages in employee motivation, retention, and attraction. In addition, researchers discovered an indirect link between intention to leave and performance-based pay. ^13^^,^^18^^,^^32^^,^^35^ According to Vroom’s expectancy theory, an employee is more likely to work harder with more incentives whether such reimbursements is correlated with personal performance. Besides, organizational performance and lower employee turnover are positively affected by incentive compensation. Hence, performance-based compensation has been acknowledged as essential HR practice. However, it is interesting that several studies report that compensation has no significant impact on OC. ^4^^,^^17^^,^^29^

*Job security.* The term “job security” relates to an employee's sense of how secure they feel in their current position. Employees quit insecure organisations. Job security creates a stable workforce, which leads to essential and pleasant outcomes. An employee is willing to utilize all of his or her skills and efforts whenever he or she feels any form of job security. As a result, an employee's intention to quit is lower. ^1^^,^^30^

### The effects of organisational commitment on turnover intention

Intentions, according to researchers, are the most direct drivers of actual turnover behaviour. ^12^^,^^23^ Lack of OC has been shown in previous studies to influence quit intentions. ^10^^,^^24^ Thus, managers must pay close attention to employees' increased OC. OC could forecast turnover intentions. ^20^^,^^21^^,^^25^ OC is one factor contributing to employees' turnover intentions, according to previous studies that looked into the components influencing turnover intention. ^6^^,^^27^

### The mediating role of organisational commitment

The literature supports the existence of a link between HR practices and turnover intention. ^7^^,^^11^^,^^16^ Employees are more likely to stay if their welfare is well-cared for by excellent HR practices. As previously said, the turnover intention is significantly connected with OC, ^5^^,^^9^^,^^14^ and it is worth investigating which measure might better predict turnover intention at this juncture. However, empirical tests conducted to support the claims are still sparse, especially in the Tehran context. Similar research was restricted to investigating the effectiveness of HRM in OC and failed to show the consequential impact on turnover intention. ^21^

Given the scarcity of research in this area, it's worthwhile considering the role of OC. We previously discussed the critical function of HR practices in managing human capital that might increase OC and reduce employee turnover intention. Therefore, we proposed OC as a mediating factor in the relationship between HR practices and turnover intention.

### Conceptual framework of the study

The sector of renewable energy (RE) is considered a knowledge-based business where human capital is uncommon and unique. However, employee turnover is a considerable issue in all fields, ^39^ including RE, which lead to difficulty finding qualified successors due to limited local expertise.

**Figure 1.**
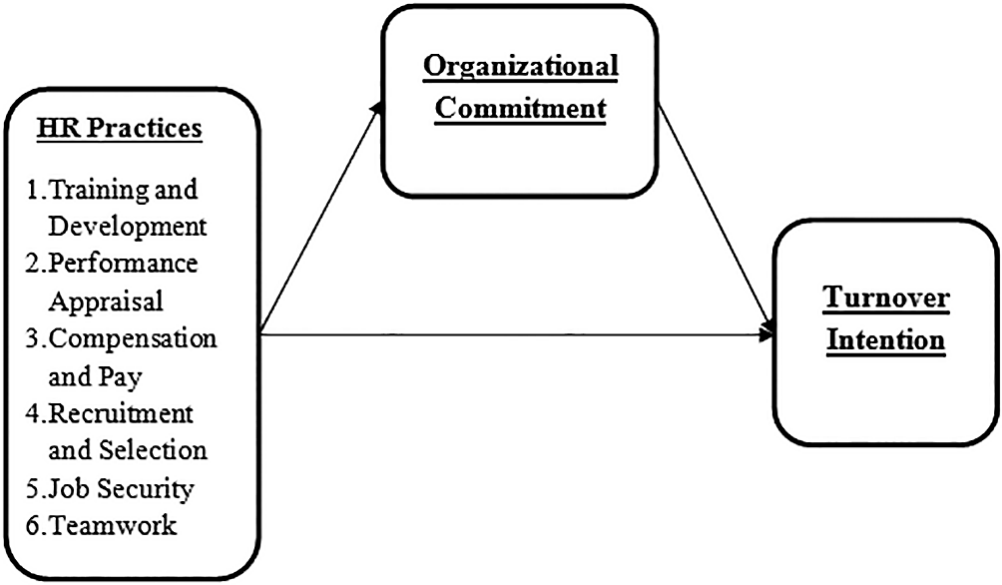
Research framework

Thus, employers are torn between nurturing talents and the risk of losing their valued employees. That is where the need arises to examine what induces turnover. Although HR practices and turnover intention have been extensively studied, ^1^ this relationship is rarely investigated with the mediating role of moral factors like OC within RE organisations. Therefore, it is imperative that employees' perceptions and interpretations of HR practices be investigated further in various situations globally. The findings are potentially valuable to employers on which HR strategies are effective in dealing with turnover. Previous studies ^7^^,^^11^^,^^16^ suggested that OC is a significant mediating factor of HRM on turnover intention. The present study aimed to explore the impact of HR practices on turnover intention regarding OC as the mediating variable in Tehran RE firms. Figure 1 presents the suggested relationships of variables in this study.

## Methods

### Ethical considerations

The study protocol was approved by the Research Ethics Committee of the Multimedia University Technology Transfer Office (Approval Number: EA0402021).

### Data collection and research instruments

Purposive sampling was used as insights was gathered from the experts in the top four leading RE companies in Tehran. The respondents must be the full time knowledgeable workers that have gone through specific trainings in this field and have been in RE industry for more than five years.

Employee information were obtained from the firms and online structured questionnaires were sent to those that met the requirements. The data was collected between November 2019 and April 2020.

The questionnaire consisted two sections ( Table 1; *Extended data*): section A surveyed HR practices and turnover intention, while section B asked about the respondents' demographic profiles. The 45-item survey employed a 5-point Likert scale ranging from 1 (strongly disagree) to 5 (strongly agree). Participants signed their consent before answering the survey, acknowledging their anonymous data to be used. The questionnaire was piloted on 30 people with similar background in Malaysia before being released to ensure face validity.

**Table 1.** Sources of questions in section B.

Variables	Items	References	Concept
**Mediating Variables**			
Training and Development	Q 1	Brownet al. (2008)	The training necessary for initial success.
	Q 2, 3	Wick and Leon (1993)	The Learning Edge - Measurement is based on organizational perspective.
	Q 4	McCann(2007)	Perception of development necessary to perform a job capably.
Compensation and Pay	Q 5, 6,7,8	Heneman and Schwabab (1985)	Pay Satisfaction Questionnaire (PSQ) – employee’s pay satisfaction.
	Q 9, 10	Strong and Harder (2009)	Fair compensation according to employee’s performance.
Job Security	Q 11, 12, 13, 14, 15	Oldham et al. (1986) and Gaertner and Nollen (1989)	Employees’ perception of their job security.
Recruitment and Selection	Q 16	Wanous (1992)	Providing a good preview of job.
	Q 17	Manton and van Es (1985)	Understanding the job and tasks by new applicants.
	Q 18, 19	Arnold and Place (2010)	Previous involvement in extension programs.
Teamwork	Q 20, 21, 22, 23, 24	Hoegl and Gemuenden (2001)	Teamwork quality into two groups: task-related and social interaction.
Performance Appraisal and Evaluation	Q 25, 26, 27	Evan (1978)	Employees’ perception of a fair performance appraisal.
	Q 28	Rousan and Henderson (1996)	Recognition of employee’s success by supervisor.
	Q 29, 30	Arnold and Place (2010)	Feedback about the evaluation process by management.
**Mediating Variables**			
Organizational Commitment	Q 31, 32, 33, 34, 35, 36, 37, 38, 39, 40	Allen and Meyer (1990)	Level of employee commitment to organization.	
**Dependent Variable**			
Turnover Intention	Q 41, 42, 43, 44, 45	Mobley et al. (1978)	The extent of employees’ turnover intentions.	

### Reliability of measurement items

The overall Cronbach's alpha values scored 0.905 (above 0.70) and hence the items were considered reliable (HR practices = 0.838; OC = 0.732; and turnover intention = 0.866).

### Normality analysis

This study used Kolmogorov-Smirnov Test to measure the normality of the data since the population size in this study is 90. Chong, Lin, Ooi and Raman (2009) stated that for data to be assumed to have normal distribution the significant level should be more than 0.05 (P value ≥ 0.05). ^38^ Table 2 shows the results of Kolmogorov-Smirnov for all variables showed a significant level of more than 0.05. Hence, normality is assumed for all variables.

**Table 2.** Tests of normality.

	Kolmogorov-Smirnov - Sig. (2-tailed)
Training & Development	0.092
Pay & Compensation	0.147
Job Security	0.166
Recruitment & Selection	0.394
Teamwork	0.249
Performance Appraisal & Evaluation	0.089
Organizational Commitment	0.421
Job Satisfaction	0.741
Turnover Intention	0.109
HR Practices	0.790

### Data analysis

The data collected was analysed with SPSS version 19 using correlational, regression and bootstrapping analyses.

## Results

### Demographic analysis

All respondents approached returned the questionnaire, leading to a 100% response rate. The majority of the participants (67.8%) were male and between 25 and 40 years old (75.6%). Employees who have worked in the same company for one to two years accounted for 28.9%. The lowest percentage (10%) worked for the same company for more than 4 years ( Table 3).

**Table 3.** Period of service of respondents.

Period of service	Frequency	Percentage
Less than 12 months	21	23.3
13-24 months	26	28.9
25-36 months	23	25.6
37-48 months	11	12.2
More than 49 months	9	10.0
**Total**	**90**	**100.0**

### Multiple linear regression results: HR practices effects on organisational commitment

Multiple linear regression analysis results confirmed the significant impact of HR practices on turnover intention (p < 0.05, R ^2^ = 0.073), with all six dimensions being statistically significant to the prediction. In order to examine the priority of the six HR practices on turnover intention, the stepwise method is used. The results suggest that all of the six dimensions of HR practices have indirect impact on the experts’ proclivity to quit their jobs. However, training and development opportunity has the most significant negative effect on turnover intention ( *β* = 0.292, Sig. = 0.005). This is followed by performance appraisal ( *β* = 0.261, Sig. = 0.013), teamwork ( *β* = 0.224, Sig. = 0.034), recruitment and selection ( *β* = 0.223, Sig. = 0.032), job security ( *β* = 0.219, Sig. = 0.038), and pay and compensation ( *β* = 0.215, Sig. = 0.041).

Also, HR practices significantly influence OC (p < 0.001, R ^2^ = 0.8) in all six dimensions. Furthermore, the impact of OC on employees’ turnover intention was significant (p < 0.05, R ^2^ = 0.057). Therefore, it is suggested that OC has a significant negative impact on employees' intention to quit RE companies.

### Bootstrapping analysis: testing mediating effect of organisational commitment

The mediating effect of OC on the relationship between HR practices and turnover intention was tested on their influence on OC using bootstrapping and path analysis.

HR practices were shown to be positively correlated with RE experts' OC (p < 0.001) (Path a). The direct effect of OC on turnover intention, however, was not significant (P _OC_ > 0.05) (Path b). The findings of path ‘c’ reveal that the effect of HR practices on turnover intention is significant (p = 0.009). Next, the results of ‘c prime’ path show that there is no significant direct effect of the HR practices on inclination to quit the job when controlling for commitment with the firm (p = 0.771). Hence, it can be concluded that there is no significant direct effect between the variables because paths ‘b’ and ‘c prime’ do not meet the requirements.

**Figure 2.**
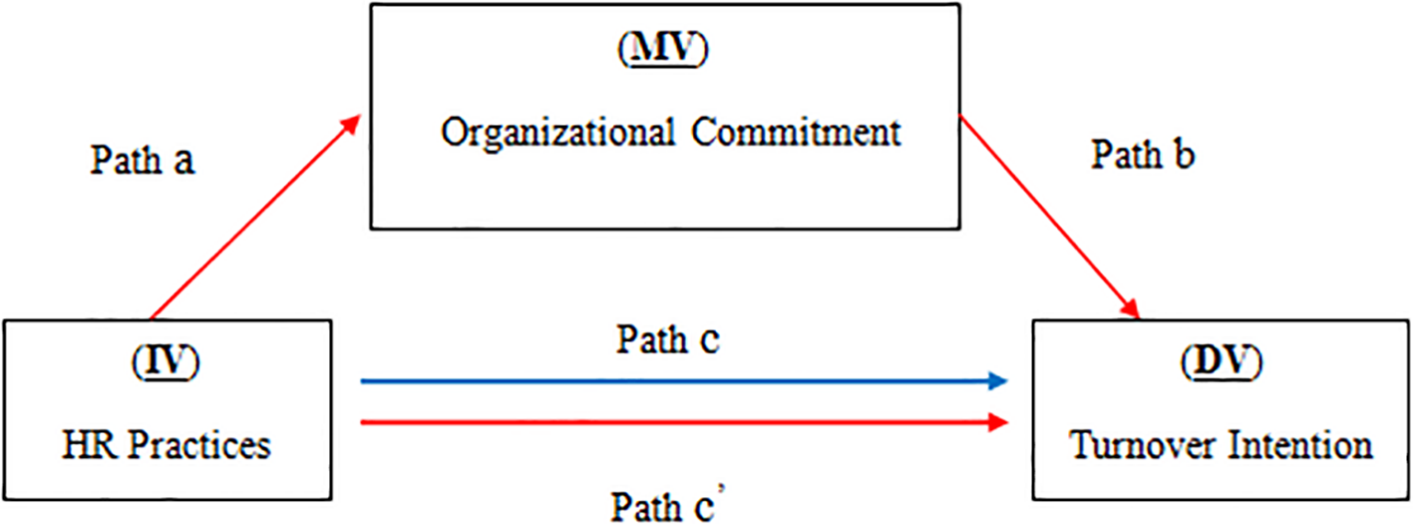
The effect of HR practices (IV) on intention to turnover (DV) with the mediating role of OC (MV).

Moreover, the results of the indirect effects of the mediating variables (Path a*b) also confirm that there is no significant effect between the variables since the zero value does occur between lower and upper values of all of the tested variables. Finally, the findings of this study also suggest that the direct effect of the mediating variable (OC) is not statistically significant ( Figure 2).

Furthermore, the indirect impacts of the mediating variables (Path a*b) indicated no significant effect between the variables, as the 0 value occurred between the lower and upper values of all of the investigated variables. Finally, the results demonstrated that the mediating variable's direct influence (OC) was not statistically significant.

## Discussion

The findings showed that HR practices positively impact OC, consistent with previous research concepts and conclusions. Employee turnover could be reduced if HR techniques are applied effectively. ^1^^,^^32^^,^^34^ Although the R ^2^ value was relatively low, the research confirmed the relevance of each of the six HR practices to turnover intention.

Training and development opportunities had the most significant indirect effect on turnover intention than other HR practices. ^22^^,^^26^^,^^29^^,^^31^ Therefore, it implied that many of the RE experts in Tehran are rather unhappy with development opportunities, leading to high turnover. However, this was not the only HR practice affecting turnover. The results also implied a need to look at the other HR practices to mitigate the turnover issue.

The finding that indicates recruitment and selection as a strong predictor of OC suggested that employees who perceive they have the skills and abilities to perform their jobs tend to have higher OC, which is in line with previous studies. ^8^^,^^22^ Therefore, the process should gauge one's potential skills and abilities and match them to the right job. Hiring an individual with matching skills and abilities would be critical in making the person a committed worker.

Compensation and pay are also strong predictors for OC. This finding contradicts the outcomes of some research done before 2000, which found no insignificant association, ^13^ and this suggested a change in causes of turnover over time. In Tehran, top managers moving for better salaries was cited as the key reason for the lack of OC. Despite their high OC, they may believe they are underpaid. Given the great demand for their skills elsewhere, they are likely to resign.

The findings validated the argument that employees' OC will be increased by exploiting sound HR practices. ^1^^,^^13^^,^^22^^,^^36^ Furthermore, all the practices predicted OC significantly to different degrees, which attested to the importance of the bundled HR practices and their relations to OC and turnover intention. More importantly, it was observed that the impact of HR practices is much more significant on OC than turnover and indicated that OC is a matter of priority before the turnover issue is addressed. These arguments are very much in support of where OC affects turnover intention. ^2^^,^^34^ Furthermore, the indirect relationship between OC and turnover intention reinforced the importance of prioritising OC, as employees with a solid commitment have a lesser tendency to leave their jobs. ^1^^,^^13^^,^^22^^,^^36^ Therefore, enhancements in HR practices are required to create and maintain a solid commitment that will retain them.

Contradicting prior studies, OC is not a significant mediator between HR practices and turnover intention. ^2^^,^^21^ Instead, it is a significant factor affected by HR practices that affect turnover intention. Nevertheless, more studies are needed to confirm the results of this study.

These findings contribute to both theory and practice. First, despite the modest findings, it contributes to the research from a theoretical aspect by looking at OC as a mediator. Nonetheless, this is a fresh perspective that has hitherto received little attention. Second, this is most certainly Iran's first analysis of the RE sector. Third, the high turnover rate justifies a timely focus on RE firms, from which practical suggestions for bettering the implementation of bundled HR practices can be derived.

The HR practices studied provide a broad guidelines to the RE companies in terms of priority in order to build greater employee commitment and to reduce their intention to leave although all of the HR practices should be enhanced at the same time in line with the concept of bundled practices. Having said so, however, management of the companies should be mindful that turnover does not depend solely on the HR practices or OC alone where there are many other factors within or beyond the control of the RE companies which must be considered. Many of the employees belong to the Generation Y category (the majority of them between the age of 25 and 35) and therefore, management must look beyond the best HR practices so that specific approaches that appeal to them are anticipated, developed, and implemented. Training and development opportunities are one of the many desires of Generation Y employees and this explain why this emerged as the most important HR practice. In addition, employees particularly Generation Y are unlikely to remain in one job for more than five years, the RE organizations need to create an environment that motivates younger employees as well as those from other generations through monetary incentives in order to enhance retention.

In addition, RE organizations must create an environment that fosters joint group actions. Asian employees in general and Generation Y employees in particular are known to work in groups to accomplish independent tasks as they use their skills, knowledge, and resources of team members to satisfy individual needs. From the recruitment perspective, the findings suggest that the RE organizations should describe the job descriptions more accurately based on the needs and characteristics and this reemphasizes the importance of a realistic job preview prior to making a selection decision. In terms of career flexibility, Generation Yers anticipate that they would change jobs frequently and therefore job security is not much of a concern to them. Although it remains as the least impactful HR practice to turnover, its significance suggests that equal emphasis is still required, particularly those of other generations. The findings also suggest on the importance of integrating other work arrangements such as work from home and work-family balance into work place. With the advent of information and communications technology (ICT), the RE companies will have little choice but to leverage on user-friendly and efficient business systems. It is common for employees to show willingness to move on to new opportunities where they perceive they will be more appreciated when their expectations are not met.

Also, it appears to be a need for companies to develop a fair and reliable evaluation method that can accurately assess employees' performance, consider their development needs, and evaluate compensation in increasing retention. ^1^^,^^13^^,^^22^^,^^36^ Furthermore, RE organisations must build a teamwork-encouraging climate and enhance the recruitment process. ^22^ These findings provide broad guidance to RE organisations regarding priority to generate higher OC and reduce turnover intention. This is critical as RE firms aim to retain personnel. Management should be aware that turnover is not solely dependent on HR practices or OC. They must look beyond basic HR practices to apply special techniques that benefit. ^36^

## Conclusion

This study established the significant effect of HR practices on OC and turnover intention. It also indicated that OC did not mediate the relationship between HR practices and turnover intention. The findings add to the growing data that HR practices should be well-managed since employees will reciprocate with more outstanding commitment and a decreased turnover intention.

These findings give essential lessons to companies that provide opportunities for career development and more autonomy in jobs might not help to reduce turnover. Instead, what is pertinent is developing HR practices that can boost OC, which subsequently reduces turnover intention.

There are several limitations in this study. Since RE is an upcoming field in Iran during the study, there are not many of such organizations where only four are known. Therefore, the population size of expert and knowledgeable employees is rather small. Although the HR practices in this study contributed 80% of the variances to OC, it is warranted for future studies to include as other potential significant HR practices. This study used the cross-sectional research design and therefore was not able to examine the long-term effect of the variables. As HR practices continue to evolve, a longitudinal research is required to validate the direction of causality involved so that a more consistent trend across time can be determined.

## Data availability

### Underlying data

Harvard Dataverse: HR Practices and Turnover Intention: The Mediating Role of Organizational Commitment in Tehran, https://doi.org/10.7910/DVN/EYNSNP. ^37^

### Extended data

Harvard Dataverse: HR Practices and Turnover Intention: The Mediating Role of Organizational Commitment in Tehran, https://doi.org/10.7910/DVN/EYNSNP. ^37^

This project contains the following extended data:

- Questionnaire sent to respondents
- A key for the readers’ interpretation
- Demographic data of participants

Data are available under the terms of the Creative Commons Zero “No rights reserved” data waiver (CC0 1.0 Public domain dedication).
