# Supplementary material for: HR practices and turnover intention; the mediating role of organizational commitment in Tehran: a cross-sectional study
Source: F1000Res. 2022 Sep 22;10:1130. Originally published 2021 Nov 8. [Version 2] doi: 10.12688/f1000research.73351.2 (PMC9582578; doi:10.12688/f1000research.73351.2)
Supplement: Peer review history

# Peer review: Reviewer response for version 2

**DOI:** 10.5256/f1000research.138505.r151156

**Published:** 2022-10-19

**Authors:** Sang Long Choi

**Affiliations:** 1 Raffles University, Johor Bahru, Malaysia

I am satisfied with the revision done on the manuscript.  I have no more comments on it. Thanks.

Is the work clearly and accurately presented and does it cite the current literature?

Partly

If applicable, is the statistical analysis and its interpretation appropriate?

Partly

Are all the source data underlying the results available to ensure full reproducibility?

No source data required

Is the study design appropriate and is the work technically sound?

Yes

Are the conclusions drawn adequately supported by the results?

Partly

Are sufficient details of methods and analysis provided to allow replication by others?

Yes

Reviewer Expertise:

Organizational Behaviour and Human Resource Management

I confirm that I have read this submission and believe that I have an appropriate level of expertise to confirm that it is of an acceptable scientific standard.

# Peer review: Reviewer response for version 1

**DOI:** 10.5256/f1000research.76996.r144168

**Published:** 2022-09-08

**Authors:** Ana Paula Moreno Pinho

**Affiliations:** 1 Department of Business, The Federal University of Ceará, Fortaleza, Brazil

The article addresses an interesting topic, but it lacks theoretical consistency and there are methodological gaps.

- The article can be improved by presenting a more consistent theoretical foundation on the commitment and HR practices.
- The bibliographic references are incomplete, it does not mention the theoretical basis used for the scales of the analyzed constructs.
- The authors do not deepen the analysis regarding the problems related to the research context.
- The authors do not present in a critical and in-depth way the analysis regarding HR practices that are shown as antecedents of the turnover intention and organizational commitment.
- The research does not present clear contributions to management practices. Also, limitations and suggestions for future research are not presented. The conclusions are very superficial.

​​​The article needs improvements in many parts: theoretical, methodological (better description of the studied context, subjects, instrument used), and a more consistent and grounded discussion based on current literature, especially on commitment.

Is the work clearly and accurately presented and does it cite the current literature?

Partly

If applicable, is the statistical analysis and its interpretation appropriate?

Partly

Are all the source data underlying the results available to ensure full reproducibility?

No source data required

Is the study design appropriate and is the work technically sound?

Yes

Are the conclusions drawn adequately supported by the results?

Partly

Are sufficient details of methods and analysis provided to allow replication by others?

Partly

Reviewer Expertise:

Human Resource Management and Organizational Behaviour .

I confirm that I have read this submission and believe that I have an appropriate level of expertise to state that I do not consider it to be of an acceptable scientific standard, for reasons outlined above.

# Response

**Published:** 2022-09-10

**Authors:** Chin Wei Chong

**Affiliations:** Multimedia University, Malaysia

Thank you for your review.

 Feedback:

- Due to the initial limitation of 2500 words, only a brief intro and review were made in the paper. In view of the diversity of the theoretical and empirical positions in this area, it is academically justified to fill the gap that suggests the need to continue conducting research that correlates human resources practices with turnover intention in different contexts, aiming to raise data that can support, refute or add elements to the fields treated here. In short, RE industry. which served as an environment for the purpose of investigating the relationship of the influence between the human resources practices adopted and mediating effect of the organizational commitment towards the turnover intention of its knowledgable employees. 
- 5 point Likert scale was used in the questionnaire. As this is the globally accepted common scaling used in survey questions, no specific reference was stated in biblio section.  Sources of items used and the definition of concepts were listed accordingly.  
- This is a correlational study that looks into the relationship between the HR practices and the turnover intention with the mediating effect of OC. Normality test, multiple regression, and mediating analysis via bootstrapping were used and explained. 
- A more detailed explanation from multiple regression analysis was added. 
- Managerial implications and limitations of the study were added. 

Thank you again for your time to review our paper.

# Peer review: Reviewer response for version 1

**DOI:** 10.5256/f1000research.76996.r144171

**Published:** 2022-08-08

**Authors:** Rashmi Kodikal

**Affiliations:** 1 Graphic Era, Dehradun, Uttarakhand, India

The Research paper is well articulated and a relevant area has been chosen for study. Organization commitment has major implications on turnover intentions and post-pandemic employee retention is a challenge across global organizations. The certain observations that I have made are as follows : 

- Details about the mode of sampling can be elaborated on. What is meant by purposive sampling can be explained from the point of view of this article?
- A deeper insight into the review of literature that validates the Six HR practices could enhance the quality of the paper. Authors are requested to do the needful.
- The discussion based on statistical evidence can be better discussed with pragmatic suggestions from the organization's point of view 
- On the whole, the paper can be indexed after incorporating the necessary changes.

Is the work clearly and accurately presented and does it cite the current literature?

Partly

If applicable, is the statistical analysis and its interpretation appropriate?

I cannot comment. A qualified statistician is required.

Are all the source data underlying the results available to ensure full reproducibility?

Yes

Is the study design appropriate and is the work technically sound?

Yes

Are the conclusions drawn adequately supported by the results?

Partly

Are sufficient details of methods and analysis provided to allow replication by others?

Yes

Reviewer Expertise:

Human Resource , Organisational Behaviour , Transactional Analysis, Services MArketing , Customer Perception

I confirm that I have read this submission and believe that I have an appropriate level of expertise to confirm that it is of an acceptable scientific standard.

# Response

**Published:** 2022-09-10

**Authors:** Chin Wei Chong

**Affiliations:** Multimedia University, Malaysia

Thank you for your review.

 Feedback:

- Purposive sampling was used as respondents need to fulfill certain requirements (expert in the field, full time, more than 5 years working experience)
-  As 2500 words is the limit of this paper, only a brief review was provided in the paper. However, a few sentences have been added in this section.
- A mode detail of managerial implications was added to strengthen the paper. 

Thank you again for your time to review our paper.

# Peer review: Reviewer response for version 1

**DOI:** 10.5256/f1000research.76996.r140041

**Published:** 2022-06-21

**Authors:** Sang Long Choi

**Affiliations:** 1 Raffles University, Johor Bahru, Malaysia

1. Need to include and elaborate on issues and problems faced in the renewable energy sector of employee turnover in the introduction section. The author also can discuss on any HR practices deficiency in this sector.
2. Kindly produce some data screening analysis for the data obtained such as normality testing and loading score for an item of each construct. 
3. Kindly show beta value or standardized coefficient value in the multiple regression results. Create on a new table to show this outcome. The reader will want to see which of these HR practices is the strongest predictor of OC and turnover intention.
4. The author needs to analyse the mediation effect of OC on each HR practice.  At the current stage, the paper uses overall HR practices as one independent variable in the mediation testing.  It is important for the reader to understand which of these practices are mediated by OC.
5. Need to elaborate on the managerial implication and research limitation in the discussion section.

Is the work clearly and accurately presented and does it cite the current literature?

Partly

If applicable, is the statistical analysis and its interpretation appropriate?

Partly

Are all the source data underlying the results available to ensure full reproducibility?

No source data required

Is the study design appropriate and is the work technically sound?

Yes

Are the conclusions drawn adequately supported by the results?

Partly

Are sufficient details of methods and analysis provided to allow replication by others?

Yes

Reviewer Expertise:

Organizational Behaviour and Human Resource Management

I confirm that I have read this submission and believe that I have an appropriate level of expertise to confirm that it is of an acceptable scientific standard, however I have significant reservations, as outlined above.

# Response

**Published:** 2022-09-10

**Authors:** Chin Wei Chong

**Affiliations:** Multimedia University, Malaysia

Thank you for your review.

 Feedback:

- The turnover issues faced in the RE industry have been added in the introduction.
- Normality analysis and scoring has been added to a new Table
- A more detailed explanation of multiple regression analysis with the value was added.
- As there is no mediation effect, no detailed explanation of each variable. However, more explanation has been added to explain the path relationship. 
- A more detailed managerial implications has been added. 

Thank you again for your time to review our paper.
